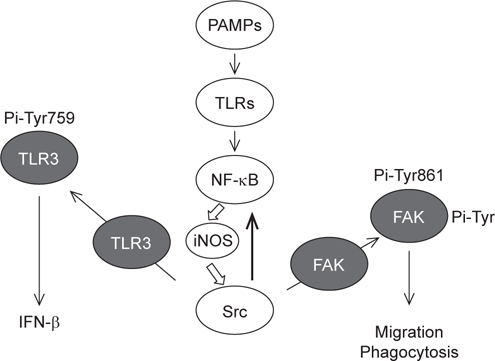# Src is required for migration, phagocytosis, and interferon beta production in Toll-like receptor-engaged macrophages

**DOI:** 10.7603/s40681-016-0014-4

**Published:** 2016-08-12

**Authors:** Ming-Chei Maa, Tzeng-Horng Leu

**Affiliations:** 1 Graduate Institute of Basic Medical Science, China Medical University, 404 Taichung, Taiwan; 2 Institute of Basic Medical Sciences, China Medical University, 404 Taichung, Taiwan; 3 Department of Pharmacology, China Medical University, 404 Taichung, Taiwan; 4 Center of Infectious Disease and Signaling Research, College of Medicine, National Cheng Kung University, 701 Tainan, Taiwan; 5 Department of Pharmacology, College of Medicine, National Cheng Kung University, 701 Tainan, Taiwan

**Keywords:** Src, Toll-like receptors, IFN-β, Macrophage activation

## Abstract

As an evolutionarily conserved mechanism, innate immunity controls self-nonself discrimination to protect a host from invasive pathogens. Macrophages are major participants of the innate immune system. Through the activation of diverse Toll-like receptors (TLRs), macrophages are triggered to initiate a variety of functions including locomotion, phagocytosis, and secretion of cytokines that requires the participation of tyrosine kinases. Fgr, Hck, and Lyn are myeloid-specific Src family kinases. Despite their constitutively high expression in macrophages, their absence does not impair LPS responsiveness. In contrast, Src, a barely detectable tyrosine kinase in resting macrophages, becomes greatly inducible in response to TLR engagement, implicating its role in macrophage activation. Indeed, silencing Src suppresses the activated TLR-mediated migration, phagocytosis, and interferon-beta (IFN-β) secretion in macrophages. And these physiological defects can be restored by the introduction of siRNA-resistant Src. Notably, the elevated expression and activity of Src is inducible nitric oxide synthase (iNOS)-dependent. Due to (1) iNOS being a NF-κB target, which can be induced by various TLR ligands, (2) Src can mediate NF-κB activation, therefore, there ought to exist a loop of signal amplification that regulates macrophage physiology in response to the engagement of TLRs.

## 1. Introduction

Rous sarcoma virus (RSV), the first identified oncogenic retrovirus, carries the *src* oncogene that encodes a 60-kDa nonreceptor tyrosine kinase [1]. Its cellular homologue (*c-src*) encodes the proto-oncogene product c-Src that acts as a co-transducer of transmembrane signals elicited from a spectrum of polypeptide growth factor receptors, including the platelet derived growth factor receptor (PDGFR) and the epidermal growth factor receptor (EGFR) [2]. Remarkably, Src knockout mice do not exhibit any obvious functional or phenotypic abnormalities except osteopetrosis [3], which is a skeletal abnormality caused by a defect in osteoclasts [4]. Osteoclasts are multinucleated cells derived from the hematopoietic precursors of the monocyte/macrophage lineage with high Src expression [5]. During bone homeostasis, osteoclasts function as resorbers of mineralized bone [6]. The absence of Src results in the impairment of bone resorption, which can be partly attributable to the suppression of osteoclast motility [6]. Despite Src being highly expressed and indispensable in osteoclasts, it’s barely detected in macrophages. This led to the speculation that Src was not involved in macrophage physiology. However, considering the close relationship between macrophages and osteoclasts, it is a reasonable assumption that Src should be pivotal in macrophage functions. Indeed, the expression of Src is inducible in macrophages exposed to various TLR ligands including lipopolysaccharide (LPS), peptidoglycan (PGN), polyinosinicpolycytidylic acid (polyI:C), and CpG-oligodeoxynucleotides (CpG) [7]. In addition to its elevated expression, the activity of Src is also greatly augmented and contributes to a diverse number of macrophage functions such as migration [7, 8], phagocytosis [9], and the secretion of IFN-β [10]. Markedly, this Src enhancement is iNOS-dependent [7, 8]. In this review, we focus on the role of the iNOS/Src axis in regulating macrophage functions.

## 2. Macrophages

Macrophages are pivotal participants in innate immunity and act as sentinels in immune responses since they can eliminate opsonized pathogens through diverse cell surface receptors and present antigens to cells to initiate adaptive immunity. In a developing embryo, the progenitors of macrophages differentiate in the yolk sac into monocytic tissue macrophages under the influence of macrophage colony-stimulating factor (M-CSF) and granulocyte macrophage colony-stimulating factor (GM-CSF). In adults, pluripotent stem cells in bone marrow develop into promonocytes (macrophage progenitors). Unlike the short-lived and non-proliferating monocyte-derived macrophages present at inflammatory sites, tissue-derived macrophages maintain their numbers through homeostatic proliferation and appear to survive for at least six weeks [11]. Resident macrophages display obvious heterogeneity in their location, cell surface markers, and function [12]. Though circulating monocytes can give rise to resident tissue macrophages, the underlying mechanisms that direct the specification of macrophages into functionally distinct subsets are still unclear. Notably, macrophages can orchestrate immune responses by inducing inflammation, which regulates both the activation and the mobilization of various immune effector cells to promote innate and adaptive immune responses. Disturbing the regulation of macrophage functions results in pathological disorders such as sepsis, autoimmune disorders, and atherosclerosis.

## 3. Toll-like receptors

The Toll-like receptors (TLRs) are a family of specialized proteins that induce protective immune responses when they detect pathogen-associated molecular patterns (PAMPs) in microbial pathogens. Toll was originally shown in Drosophila as an essential receptor for host defense against fungal infection [13]. Later, a mammalian homologue of the Toll receptor (now termed TLR4) was demonstrated to induce inflammatory responses [14]. Certain TLRs (*i.e*. TLR1, 2, 4, 5, 6 and 11) are found on the cell surface, while others (*i.e*. TLR3, 7, 8 and 9) are detected almost exclusively in intracellular compartments such as endosomes [15, 16]. TLR2 detects peptidoglycan (PGN), a major bacterial cell wall component. TLR3 recognizes viral double-stranded RNA [17]. TLR4 acts as a signaling receptor for lipopolysaccharide (LPS), an outer membrane component of Gram-negative bacteria [18, 19]. TLR9 senses the unmethylated CpG-oligodeoxynucleotides (CpG) that are frequently found in bacteria, but not in vertebrate DNA [20]. Subsequent to recognition of a PAMP, TLR will recruit a variety of adaptors, including TRIF, MyD88, TRAM, and TIRAP/Mal. It is well-established that TLR9 requires MyD88, that TLR3 utilizes TRIF, that TLR2 needs MyD88 and TIRAP, and that TLR4 uses all four of the aforementioned adaptors [21]. Through individually preferential adaptors, TLR engagement triggers downstream signaling pathways that activate NF-κB or MAP kinase, which in turn produce proinflammatory cytokines required for host defensive strategies [16].

## 4. The Src family kinases

The Src family kinases (SFKs) is a family of kinases that play key roles in regulating signal transduction by various cell surface receptors in the context of a diversified cellular environment. Src is the prototypic member of SFKs that comprises Src, Yes, Fyn, Lck, Lyn, Fgr, Hck, Blk, and Yrk [22]. While Src, Yes, and Fyn are ubiquitously expressed, the others are more selectively expressed in hematopoietic cell lineages. Because of alternative splicing or the use of alternative start codons, several SFKs exhibit multiple isoforms. Structural similarity among the SFKs can be revealed by aligning their amino acid sequences. The conserved regions include: (1) the N-terminal myristoylation signal that mediates the attachment of SFKs with the plasma membrane; (2) the SH3 and SH2 domains that are responsible for direct protein-protein association; (3) the kinase domain; (4) the C-terminal regulatory domain. Tyr416 and Tyr527 located within the kinase domain and the C-terminal regulatory region respectively are two important phosphorylation sites. While the phosphorylation of Tyr416 is self-mediated, the phosphorylation of Tyr527 is mediated by CSK (C-terminal Src kinase) that downregulates SFK activity. According to X-ray crystallography analyses and mutational studies, Src has been proposed to be held in an inactive conformation by the intra-molecular interaction between Pi-Tyr527 and SH2 as well as the SH2/kinase linker and SH3 [23]. Src becomes active when these associations are disrupted.

### 4.1. Constitutive expression of myeloid-specific Src family kinases

Src, Yes, and Fyn can be detected in most tissues. In contrast, the other members of the SFK family are distributed mainly in cells of hematopoietic lineage [24]. Fgr, Hck, and Lyn are myeloidspecific SFK members that are predominantly expressed in macrophages. Given the development of the tumoricidal activity of LPS-stimulated murine peritoneal macrophages (PEMs) and the release of eicosanoid mediators from LPS-stimulated RAW264.7 macrophages are suppressed by herbimycin A (a tyrosine kinase inhibitor) [25], and considering TLR4 lacks an intrinsic tyrosine kinase activity, Fgr, Hck and Lyn seem to be the major players responsible for LPS-elicited tyrosyl phosphorylation and macrophage activation. Astonishingly, the full LPS responsiveness retained by the PEMs and the bone marrow-derived macrophages (BMDMs) derived from mice deficient of Fgr, Hck, and Lyn [26], implicates these three myeloid SFKs as being dispensable for LPS-induced macrophage activation. In the meantime, an intriguing question is also raised as to what the identity of the kinase that mediates tyrosyl phosphorylation required for the LPS-exerted effects in macrophages is.

### 4.2. Inducible expression of Src

In a previous time-course study, we observed that in addition to immediate and transient activation, SFKs also exhibited sustained and long-lasting activity that was speculated to be crucial for LPS-mediated responses in macrophages [27]. However, the intact LPS responsiveness observed in PEMs and BMDMs from *fgr* ^*-/-*^ *hck* ^*-/-*^ *lyn* ^*-/-*^ mice implies that Fgr, Hck, and Lyn are not obligatory for macrophage activation. Due to the expression of the non-myeloid SFKs being barely detectable in resting macrophages, one might speculate that the myeloid SFKs, with their high expression, perform the house-keeping job while the expression and activity of the critical non-myeloid SFK(s) should be induced for macrophages to defend pathogenic invasions. Considering that Src is indispensable for the resorbing activity of macrophage-related osteoclasts [28], it is therefore likely to be the long-sought SFK responsible for LPS-evoked macrophage activation. Indeed, LPS enhances the expression of Src in both PEMs and RAW264.7 macrophages in a timedependent manner [8]. Similar upregulation of Src in PEMs recovered from LPS-challenged rats further indicates its physiological significance [8, 27]. Intriguingly, Src induction is also detected in PEMs, BMDMs, and RAW264.7 macrophages treated with CpG-oligodeoxynucleotides (CpG, TLR9 ligand), peptidoglycan (PGN, TLR2 ligand), or polyinosinic-polycytidylic acid (polyI:C, TLR3 ligand). This CpG-, LPS-, PGN-, and polyI:C-induced Src expression can be attributed to the increased level of the src transcript [7]. Notably, the expression of Fgr, Hck, and Lyn are almost unaltered in PEMs, BMDMs, and RAW264.7 macrophages exposed to various TLR ligands [7]. A mechanistic study in TLRactivated macrophages revealed that a pharmacological blockade or a knockout of inducible nitric-oxide synthase (iNOS) hampers Src enhancement. Remarkably, either a NO donor (*i.e*. SNAP) or a cGMP analogue (*i.e*. 8-Br-cGMP) was observed to restore Src expression in iNOS-null PEMs, indicating the participation of NO/cGMP in Src induction elicited by diverse TLR ligands. This inducible characteristic of Src suggests its critical role in relaying signals in macrophages in response to TLR engagement. To date, the requirement of Src in macrophage physiology at least includes migration, phagocytosis, and IFN-β production.

## 5. Src and macrophage migration

Macrophages exhibit increased motility when encountering TLR ligands [7]. Notably, this process is PP2-sensitive, indicating the involvement of SFKs. In contrast to the almost unaltered expression of the myeloid SFKs, the enhanced expression of Src mediated by activated TLRs prompts its importance in macrophage movement. Indeed, Src knockdown has been observed to lead to suppressed CpG-, LPS-, PGN-, and polyI:C-elicitede motility in RAW264.7 macrophages, and ectopically expressed avian Src restored this defect [7, 8]. Focal adhesion kinase (FAK), a downstream target of Src, can regulate focal adhesion turnover and migration in fibroblasts [29]. Src mediates FAK Pi-Tyr861, an indicator of FAK activation [30]. Macrophages devoid of FAK display mobility defects that coincide with increased protrusive activity at the cell periphery, decreased adhesion turnover, and an inability to form stable lamellipodia for directional migration [31]. Consistent with the elevated FAK Pi-Tyr861 in CpG-, LPS-, PGN-, and polyI:C-treated RAW264.7, FAK-deficient macrophages exhibit impaired migration in response to various TLR ligands. Of note, concordant with its constant expression in TLR engaged macrophages, Lyn attenuation does not hamper macrophage mobility. Given that Src is NO- and cGMP-inducible, simultaneously augmented Src expression, elevated activity of Src and FAK as well as cell movement are observed in macrophages exposed to SNAP and 8-Br-cGMP. Furthermore, the suppressed CpG-, LPS-, PGN-, and polyI:C-evoked motility in iNOS-null macrophages can be rescued by SNAP and 8-Br-cGMP [7]. These findings corroborate that the NO/cGMP pathway contributes to Src induction and macrophage mobility *via* TLR ligands.

## 6. Src and macrophage phagocytosis

Phagocytosis is a phylogenetically conserved process that is pivotal for innate immunity. Through a spectrum of phagocytic receptors (*i.e*. Fcγ receptors and complement receptor 3) and TLRs, macrophages detect the presence of various pathogens in the body [32, 33]. Engagement of these receptors triggers the activation of a series of intracellular signaling pathways that lead to membrane trafficking as well as dynamic and rapid cytoskeletal rearrangements that are required for macrophage phagocytosis. Interestingly, reduced Src expression and impaired phagocytosis are observed simultaneously in LPS-treated PEMs from C3H/NeJ mice (with defective TLR4) as compared to those from C3H/HeN mice (with wild type TLR4) [9]. This finding suggests that LPS-mediated Src expression is TLR4-dependent and Src participates in LPS-induced phagocytosis. Indeed, Src attenuation hampers LPS-evoked phagocytosis and FAK Pi-Tyr861, which can be rescued by ectopic Src. Consistent with the involvement of FAK in integrin-mediated macrophage phagocytosis of *Yershinia pseudotuberculosis* [34], FAK attenuation diminishes the uptake of GFP-*E*. *coli* in LPS-treated macrophages [9]. In contrast, the knockdown of Lyn does not affect this LPS-triggered event.

## 7. Src and macrophage interferon-beta (IFN-β) production

Type I interferon (IFN-I) comprises the IFN-α family and IFN-β and exerts a wide spectrum of biological functions including the inhibition of viral replication [35]. In addition to antiviral activity, IFN-α/β also regulates the homeostatic differentiation of natural killer cells, dendritic cells, B cells, T cells, and osteoclasts [36, 37]. Double-stranded RNA (dsRNA) induces phosphorylation of TLR3 and subsequently ignites signaling pathways to produce IFN-β. The expression of IFN-α/β is primarily regulated by multiple transcription factors such as HMG1(Y), NF-κB, AP1, and IRFs [38]. Activation of IRF3 and IRF7 promotes *ifn* gene transcription. Phosphorylation of TLR3 at both Tyr759 and Tyr858 independently mediates PI3K and TBK1 activation, leading to the phosphorylation and activation of IRF3 [39]. A biphasic (early *versus* late) TLR3 Pi-Tyr759 has been observed in dsRNA-stimulated macrophages. Src can directly phosphorylate TLR3 Tyr759 *in vitro* and *in vivo*. Markedly, Src-mediated late TLR3 Pi-Tyr759 leads to the nuclear accumulation of IRF3/IRF7 and the increase of IFN-β production. Also of note, *via* the down-regulation of Src, dsRNA-elicited TLR3 Pi-Tyr759, the nuclear accumulation of IRF3/IRF7, and IFN-β generation are inhibited in PEMs devoid of iNOS. Strikingly, TLR3 knockdown destabilizes Src and decreases the nuclear level of IRF3/IRF7 and IFN-β secretion in macrophages exposed to LPS, which is known to enhance Src and IFN-β expression [10]. Thus, there exists a “crosstalk” between TLR3 and TLR4, a communication which is Src-dependent and occurs in the TLR3-containing endosomes. Engaged TLR4 induces iNOS and Src expression, which leads to the complex formation between TLR3 and Src, an event that stabilizes Src and increases the following TLR3 Pi-Tyr759. However, Src induction, but not *ifn-β* transcription, is restored in dsRNA- or LPS-treated macrophages expressing 759F-TLR3, TLR3 Pi-Tyr759 seems not to be required for Src stabilization but plays a critical role in IFN-β generation [10]. Concurrent with the dispensability of Fgr, Hck, and Lyn for LPS/TLR4 signaling in macrophages, the depletion of any of the three myeloid SFKs does not affect TLR3 Pi- Tyr759. Moreover, Lyn knockdown does not suppress dsRNA-evoked *ifn-β* transcription and IFN-β secretion. It is noteworthy that FAK is involved in TLR-mediated macrophage migration and phagocytosis, but not in dsRNA-triggered IFN-β production in RAW264.7 macrophages.

## 8. Conclusions and future perspectives

Unlike the large repertoire of rearranged receptors utilized by T and B cells in adaptive immunity, innate immunity detects microorganisms *via* limited germline-encoded PAMP-recognition receptors including TLRs. Irrespective of their utilization of different TIR-containing adaptors and their different localization, engaged TLRs activate NF-κB, and augment the expression of iNOS and proinflammatory cytokines [19]. Given that the aforementioned TLRs are located on either plasma membranes (*i.e*. TLR2 and TLR4) or endosomes (*i.e*. TLR3 and TLR9), and their mediated signaling pathways can be divided into MyD88-independent (*i.e*. TLR3) and -dependent (*i.e*. TLR2, TLR4 and TLR9) pathways, the iNOS-mediated upregulation of Src in response to various TLR engagements seems to be a general mechanism for diverse macrophage functions, including migration, phagocytosis, and IFN-β production. A simple model illustrating the responsible mechanism for TLR-triggering, Src-dependent migration, phagocytosis, and IFN-β production in macrophages is proposed in Figure 1. Given (1) Src is critical for the recruitment of macrophages and the progression of chronic inflammation; (2) Src is indispensable for phagocytosis and bacterial killing in LPSexposed macrophages; and (3) Src mediates TLR3 Pi-Tyr759, which is required for IFN-β production, therefore the potential of Src to be the therapeutic target of a spectrum of inflammatory and infectious diseases can be highlighted. Strikingly, constitutive activation of Src results in TLR3 Pi-Tyr759 and IFN-β secretion in v-Src-transformed cells. It has been well-established that type I IFN possesses antiproliferation activity in cancer cells, thus TLR3 is expected to play a negative role in cancer cell growth. However, *via* association with Src, TLR3 increases Src stability and contributes to its mediated anchorage independent growth. Because of these results, TLR3 might be a potential target for anti-cancer therapy.

**Figure 1:**